# Evaluation of Interactions Between Thyroid Dysfunction in End-Stage Renal Disease Patients: A Cross-Sectional Study

**DOI:** 10.7759/cureus.35088

**Published:** 2023-02-16

**Authors:** Shobhit Shakya, Satish Kumar, Virendra Verma, Harish Gupta, Satyendra Kumar Sonkar, Virendra Atam

**Affiliations:** 1 General Medicine, Dr. Ram Manohar Lohia Institute of Medical Sciences, Lucknow, IND; 2 Medicine, King George Medical University, Lucknow, IND; 3 General Medicine, Rajarish Dashrath Autonomous State Medical College, Ayodhya, IND; 4 Internal Medicine, King George Medical University, Lucknow, IND

**Keywords:** prevalence, subclinical hypothyroidism, gfr, ckd, thyroid

## Abstract

Background: Thyroid hormones have a very crucial role in the regulation of metabolism, synthesis of proteins, development, and influencing functions of various other hormones in the human body. While both kidneys play an essential role in the metabolism of thyroid hormone by conversion of thyroxine (T4) to triiodothyronine (T3). In patients with chronic renal failure, frequent abnormal thyroid functions are observed.

Aims: To evaluate thyroid function in patients of chronic renal failure and to find out their correlation with the severity of the disease.

Methods: A total of 192 patients were selected for the study after applying inclusion and exclusion criteria. A thyroid function test was done in all enrolled subjects. Serum estimation of T3, T4, and thyroid stimulating hormone (TSH) was done by the chemiluminescent immunoassay (CLIA) method, urea was estimated by the diacetyl monoxide method (DAM, Method), and serum creatinine by Jaffe's method. The results were evaluated for age, sex, and estimated glomerular filtration rate (eGFR) of the patients in view of thyroid dysfunction.

Results: Of all 192 patients enrolled in the study, 124 (64.58%) were male and 68 (35.41%) were females. The observed male-to-female ratio was 1.93:1.18. The mean age of the study group (mean +/- standard deviation, SD) in males was 42+/-18 and in females 38+/-11 years (p value = 0.258). Significant reductions of serum T3, T4, and elevation of TSH were noted in both sexes. A reduced level of T3 was observed in 38.54% (42 males and 32 females) patients, reduced T4 in 34.37% (42 males and 22 females) patients, and subclinical hypothyroidism (SCH) in 16.7% (12 males and 20 females) patients. Biochemical overt hypothyroidism was noted in 7.29% (six males and eight females) of patients.

Conclusion: Chronic renal failure is a condition of thyroid hypofunction. A higher prevalence of SCH and clinical hypothyroidism is reported here in chronic kidney disease (CKD) patients. The severity of thyroid hypofunction increases with a progressive reduction in eGFR. Hypothyroidism in CKD patients may be due to different onset mechanisms other than anti-thyroid antibodies.

## Introduction

The thyroid gland regulates most of the physiological actions of the body. Both the kidneys and thyroid are physiologically and functionally related to each other [1]. Now chronic kidney disease (CKD) has been known to alter the pituitary-thyroid axis along with the peripheral thyroid hormones metabolism. Various researchers have observed thyroid dysfunctions like hypothyroidism, hyperthyroidism, and euthyroid state in renal failure patients. The thyroid hormone plays a major role in the growth and development of the kidneys while the kidneys did metabolism, degradation, and excretion of this hormone [1-2].

As iodine clearance takes place in glomerular filtration in the kidneys that is the reason in cases of chronic renal failure, iodide excretion is diminished, leading to higher plasma concentration of inorganic iodide and hence its uptake [1]. A higher concentration of inorganic iodide inside the body may significantly inhibit the formation of thyroid hormones by disturbing the pituitary-thyroid axis and peripheral metabolism of thyroid hormones (Wollf-Chaikoff effect). This mechanism is responsible for hypofunction of the thyroid gland in chronic renal failure patients. Thyroid disorders are also linked with glomerulonephritis most commonly by a common autoimmune cause [2]. Thyroid and renal malignancies associations have also been reported. So, there is a common association between renal failure and thyroid disorders [2]. A fall in the estimated glomerular filtration rate (eGFR) in these patients makes a higher possibility of the development of hypothyroidism [3]. Various researchers reported a prevalence of thyroid hypofunction of 13% in early phases of renal failure to 70% in end-stage renal disease (ESRD) patients [4-7]. Uremia is one of the crucial links between thyroid dysfunction and CKD. Thyroid disease, treated cases have a decreased chance of occurrence of renal dysfunction. Renal dysfunctions have been reported to coexist with specific altered values of thyroid hormones [6-7]. Current research is conducted to simplify the importance of correlations between kidney disease and thyroid functions. This knowledge is important as it predicts a link between two separate entities. 

## Materials and methods

Study design and setting

**Figure 1:**
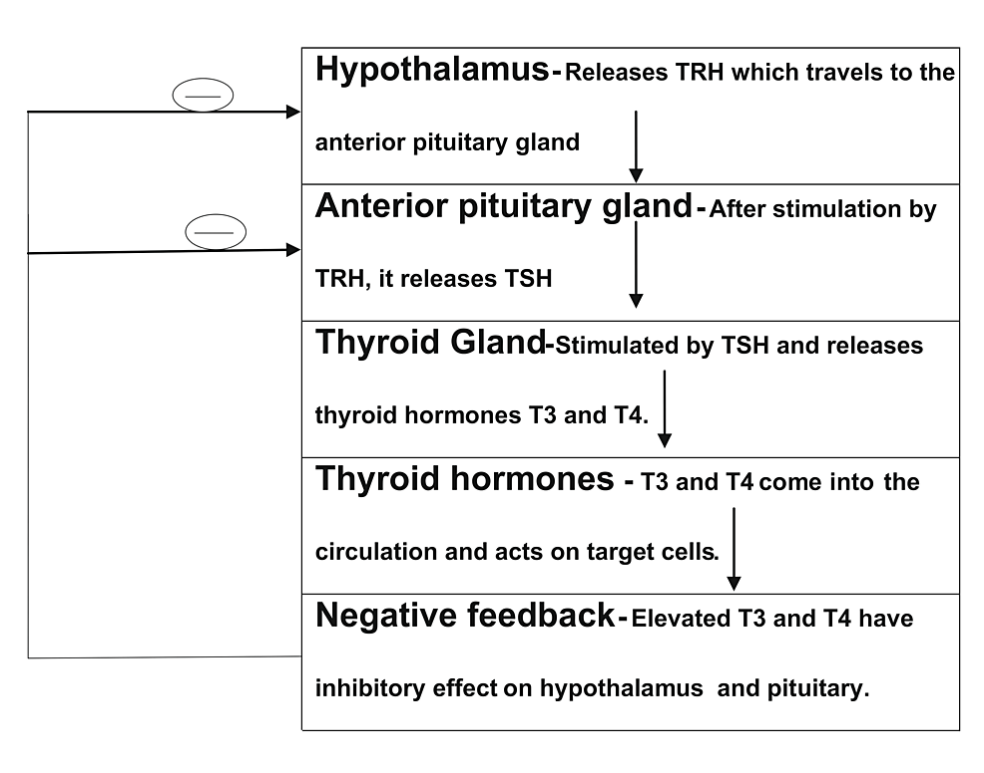
Depicting normal thyroid gland physiology. T3, tri-iodothyronine; T4, thyroxine; TSH, thyroid stimulating hormone; TRH, thyroid releasing hormone

**Figure 2:**
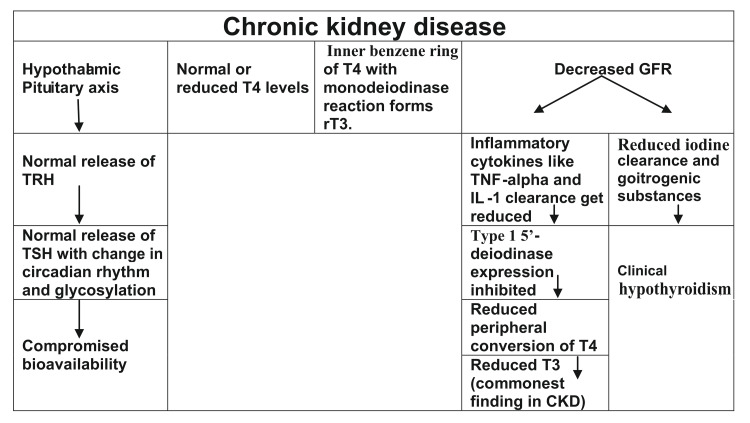
Depicting relations of CKD to thyroid dysfunction. T3, tri-iodothyronine; T4, thyroxine; TSH, thyroid stimulating hormone; TRH, thyroid releasing hormone; GFR, glomerular filtration rate; IL-1, interleukin 1; TNF, tissue necrosis factor; CKD, chronic kidney disease

**Figure 3:**
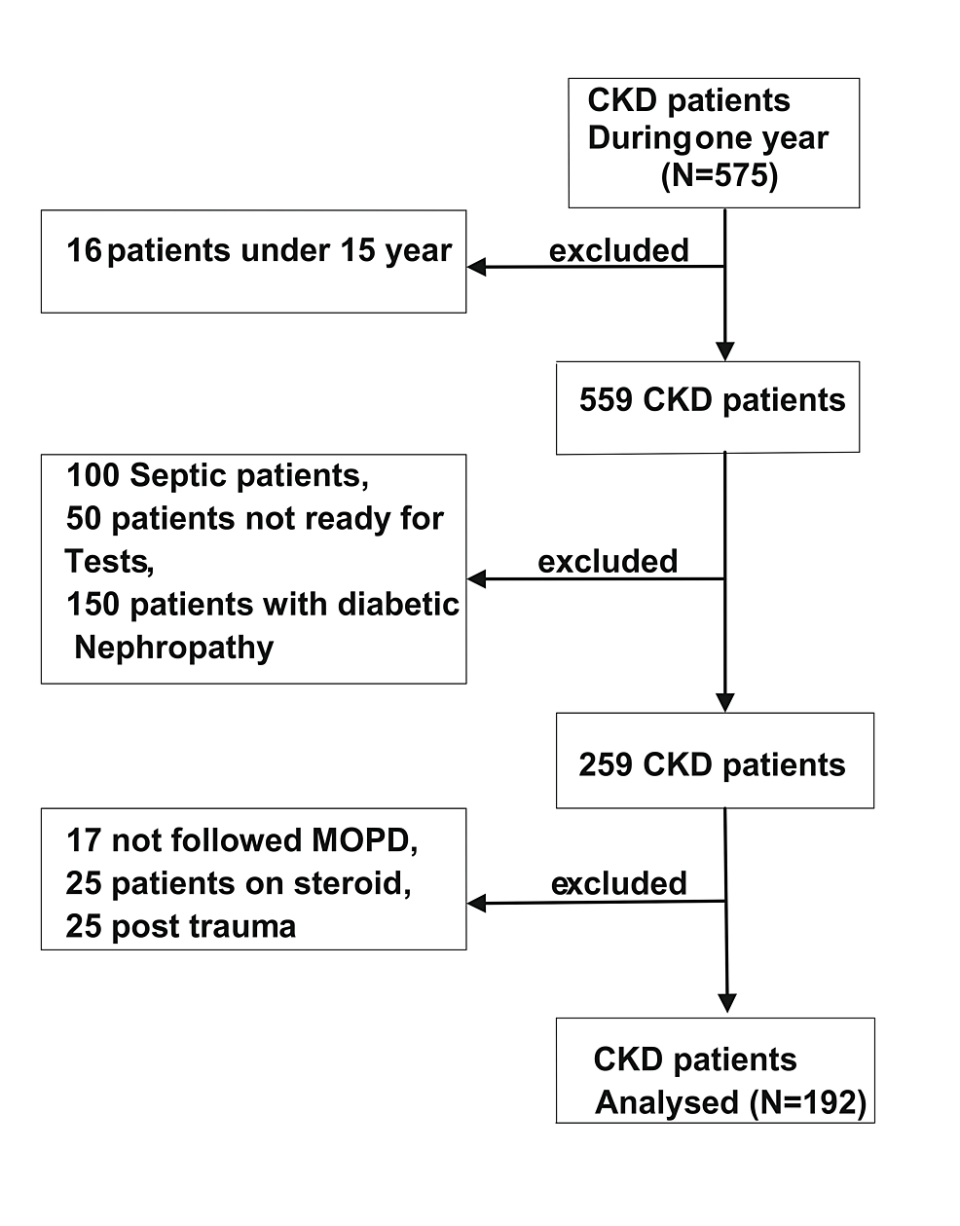
CONSORT flow diagram. CKD, chronic kidney disease; MOPD, medicine outpatient department

The present study is a hospital-based cross-sectional study done for a period of one year (from November 2021 to October 2022) in the Department of Medicine at King George’s Medical University Lucknow. CKD patients attending our medicine outpatient department (MOPD) were investigated for thyroid and kidney function for overt hypothyroidism, subclinical hypothyroidism (SCH), and alteration of tri-iodothyronine (T3) and thyroxine (T4). The total number of CKD patients during this period was 575, but after applying exclusion criteria, a total of 192 patients were selected for the study. Of all, 68 were female and 124 were male patients. Patients having features or history of acute illness, thyroid dysfunction, thyroidectomy/thyroid disorder recent surgery, drugs altering thyroid profile (like amiodarone, phenytoin, steroids, estrogen pills, beta-blockers, iodine-containing drugs, nephrotic syndrome, children's, antenatal check-ups (ANC) patients, diabetic nephropathy, trauma, and burns were excluded from the study. All the enrolled cases were followed up by a medical examination according to pre-prepared proforma (Figures 1-3).

Laboratory investigations

After 12 h of fasting, an early morning blood sample was taken from each subject and kept standing to clot. Serum was separated and analyzed after centrifugation. Serum estimation of total T3, total T4, and TSH was done by the chemiluminescent immunoassay (CLIA) method; urea by diacetyl monoxide method (DAM, Method), and serum creatinine by Jaffe's method were estimated. The expected normal range of total T3, 70-204 ng/dL, total T4, 4.5-11 µg/dL, and TSH, 0.5-4.5 mU/L were considered. Laboratory values beyond this range were expected to have thyroid dysfunction. Every CKD patient was classified into different five stages of CKD by measuring the eGFR and urinary protein.

Assessment of thyroid and kidney functions

The eGFR was calculated by using the Modification of Diet in Renal Disease (MDRD) formula: (eGFR in mL/min/1.73 m2) = 186 x (creatinine/88.4) - 1.154 x (age) - 0.203 x (0.742 if female) x (1.210 if black). CKD staging was done by using Kidney Disease Improving Global Outcome (KDIGO) criteria.

Statistical analysis

The collected data of all 192 enrolled subjects were fed into an excel spreadsheet. We did a descriptive analysis of the collected data. Data were statistically analyzed by the Kruskal-Wallis equality of population rank test, Pearson's chi-square test, and Mann Whitney test using SPSS and Microsoft excel. A value of p < 0.05 was considered statistically significant.

## Results

The research group comprised 192 subjects with 124 (64.58%) males and 68 (35.41%) females. The male-to-female ratio was observed at 1.93:1.18 and the mean age of the study population (mean +/- standard deviation, SD) in males was 42+/-18 and in females 38+/-11 years (p=0.258) (Table 1). It was estimated that the mean value of T3 in males was 62+/-21.4 and in females, it was 56.8+/-20.8 ng/dL (p=0.35) (Table 2). On comparing the mean of T3 among various age groups, it was found that the mean in patients with <30 years was 65.14+/-17.7 ng/dL, in patients in the age group of 31-60 years it was 58.8+/-24 ng/dL, and in age group >60 years it was 56+/-16 ng/dL (p=0.448) (Table 3).

**Table 1:** Age and sex-wise distribution of cases. SD, standard deviation

Sex	<30 years	31-60 years	>60 years	Mean+/-SD
Male	44	54	26	42+/-18
Female	14	52	2	38+/-11

**Table 2:** Comparison of the level of T3 between males and females. p=0.204; T3, triiodothyronine

T3 level (ng/dL)	Male (n)	Female (n)
<60	42	32
60-200	82	36

**Table 3:** Mean of T3 and T4 among various age groups. SD, standard deviation; T3, triiodothyronine; T4, thyroxine

Age in groups	Number of patients	T3 Mean =/- SD ng/dL (p=0.448)	T4 Mean =/- SD ng/dL (p=0.503)
<30	58	65.14+/-17.7	5.6+/-2
31-60	106	58.8+/-24	5.7+/-2.6
>60	28	56+/-16	4.86+/-1.6

On the basis of eGFR, two separate groups were created in this study; of which there were a maximum of 116 people (60%) with T3 between 60 and 200 ng/dL having mean GFR 5.1+/-3.9 mL/min/1.73 m2 SD, 76 (39.58%) with T3 <60 ng/dL having mean GFR 3.9+/-1.7 mL/min/1.73 m2 SD (Mann Whitney test p=0.07).

A reduced level of T4 (<4.5 ug/dL) was seen in 22 (32%) females, normal level (4.5-11 ug/dL) of T4 was observed in 44 (64%) females while one female was having high value (>11 ug/dL) of T4. A low level of T4 was also observed in 42 (33%) males while a normal level of T4 was observed in 82 (66%) males (p=0.397).

The calculated mean of T4 in females was 5.49+/-2.3 while in males, it was 5.65+/-2.4 (p=0.63). We compared the mean of T4 among the three age groups. Subjects with age <30 years were having mean of T4=5.6+/-2, age group 31-60 years have a mean of T4=5.7+/-2.6 and >60 years had a mean of T4=4.86+/-1.6 (p=0.503). On the basis of eGFR three different groups were created in this study; of which there was a maximum of 124 people (64.58%) with T4 between 4.5 and 12 ug/dL have mean GFR 4.9+/-3.7 mL/min/1.73 m2 standard deviation (SD), 64 people (33.33%) with T4 < 4.5 ug/dL have mean of 4.38+/-2.1 mL/min/1.73m2 SD and one subject with T4 > 12 ug/dL have mean of 3.00+/-0 mL/min/1.73 m2 SD (p=0.63).

The TSH was compared between male and female subjects and it was observed that a low level of TSH was seen in two females (2.94%), a normal level of TSH was seen in 38 females (55.8%) while SCH was reported in 20 females (29.4%) and overt hypothyroidism was depicted in 8 females (11%). In male patients the trend was different. A reduced value of the thyroid was observed in six subjects (4.84%), the normal value of TSH in 12 males (9.6%) while overt hypothyroidism in six males (4.84%) was seen. Here the p-value was significant (p=0.035). There was a significant difference in TSH in males and females. We conclude that SCH was prevalent in females as compared to males. The calculated mean value of TSH in males was 3.0+/-3.0 and in females mean was 5.9+/-7.6 (p=0.11). A total of 192 patients were staged into five categories of CKD with 44.32% in stage five, 20.22% in stage four, 23.19% in stage three, 10.31% in stage two, and 1.96% in stage 1. There was no significant difference (p=0.35) in TSH value in various age groups. There was also no significant difference (p=0.175) in the mean value of GFR in various values of TSH (Tables 4-6).

**Table 4:** Mean of eGFR between various levels of T3. SD, standard deviation; T3, triiodothyronine; GFR, glomerular filtration rate

T3 (ng/dL) p=0.07	Number of patients	Mean of GFR (mL/min/1.73m2)=/-SD
<60	74	3.9+/-1.7
60-200	116	5.1+/-3.9

**Table 5:** Mean of eGFR between various levels of T4. SD, standard deviation; T4, thyroxin; GFR, glomerular filtration rate

T4 (ug/dL) p=0.63	Number of patients	Mean of GFR (mL/min/1.73m2)=/-SD
<4.5	64	4.38+/-2.1
4.5-12	124	4.9+/-3.7
>12	2	3.00+/-0

**Table 6:** Mean of eGFR between various levels of TSH. SD, standard deviation; GFR, glomerular filtration rate; TSH, thyroid stimulating hormone

TSH (u IU/mL) p=0.175	Number of patients	Mean of GFR (mL/min/1.73m2)=/-SD
<0.3	8 (6 Male+2 Female)	4.75+/-2.5
0.3-5.5	136 (100 Male+38 Female)	4.5+/-3.6
5.5-10	32 (12 Male+20 Female)	4.81+/-2.3
>10	14 (6 Male+8 Female)	5.58+/-1.6

## Discussion

The CKD alters the secretion of various hormones in the human body and till now this is not known to us as to what extent these alterations lead to the uremic syndrome. Most CKD patients have thyroid dysfunction. Thyroid autoimmunity and subclinical primary hypothyroidism are common occurrences in CKD patients who do not need long-term dialysis [8]. Induction of kidney disease in hypothyroidism may be due to decreased renal blood flow, reduced GFR, and tubular dysfunction [9-10]. The present study was done to search out the prevalence of thyroid dysfunction in CKD patients and its correlation with the severity of the disease.

The findings in prior research on the correlation of thyroid disturbances in CKD have been different. As hemodialysis (HD) changes the thyroid profile, CKD patients with HD were excluded from the study. A study done by Chandra, in 2016, reported SCH and clinically apparent hypothyroidism in up to 40% and 16%, respectively, who were on conservative management [11]. In the current study, there was a significant decrease in serum T3 level, T4 level, and elevation of TSH level in male and female cases. A low level of T3 was observed in 38.54% (42 males and 32 females) of patients, a low level of T4 was observed in 34.37% (42 males and 22 females) of patients, SCH was observed in 16.7% (12 male and 20 females) patients and biochemical overt hypothyroidism was also observed in 7.29% (six males and eight females) of CKD patients. However, SCH was more prevalent in females (29%) as compared to males (9.6%) with a significant p-value (0.035). The present study differs from prior studies by observing a prevalence of SCH and clinical hypothyroidism. A higher prevalence of subclinical and clinical primary hypothyroidism in patients with decreased estimated GFR (eGFR) was seen which was independent of age and sex. Similar findings were also reported by Chonchol et al. [12].

This study included a total of 192 patients, of which 46 were having thyroid dysfunction, 32 patients were having SCH, and 14 patients having clinical hypothyroidism. Here the absolute prevalence of hypothyroidism was more in the lower GFRs in comparison to other studies. Most of the cases in the present research fell in the CKD final stage; it may be due to the reduced GFR of referred patients to this tertiary care center. Higher TSH values were observed with increasing age [13-14]. The mean age (mean +/- SD) of the study population was 42+/-18 and 38+/-11 years (p=0.258) in males and females respectively.

Of all 192 total patients, 14 were found to be clinically hypothyroid with a higher number in Stage V (45%), while in Stage II, it is lowest (12%). Lo et al. observed that the prevalence of hypothyroidism was more with a decrease in GFR occurring in 5.4% of cases, with GFR ≥90, 10.9%, with GFR 60-89, 20.4%, with GFR 45-59, 23.0%, with GFR 30-44, 23.1% and with GFR <30 (p < 0.001 for trend) [14-15]. The overall prevalence of SCH was 17%. In a study of the year 2008, of all 3089 adult patients, 293 (9.5%) were having subclinical primary hypothyroidism and 277 (9%) with an eGFR <60 mL/min/1.73 m2 [12]. Although a number of hypotheses explain the various contributing factors for thyroid dysfunction in CKD patients, such as low circulating thyroid hormone levels, insufficient binding to carrier proteins, decreased tissue thyroid hormone content, alteration in iodine metabolism, autoimmune thyroiditis, and reduced peripheral sensitivity to hormones, the exact underlying pathomechanisms connecting advanced stage CKD and primary hypothyroidism were still unknown to us [16-18]. Treatment of subclinical and overt hypothyroidism in CKD patients by thyroid hormone replacement therapy (THRT) may improve the blood flow to the kidneys and hence its functions [19].

Limitations of the study

There are several limitations of the study. First, the thyroid function test was not investigated in all patients as some denied testing. Second, we, did not assess serum anti-thyroid peroxidase antibodies, and anti-thyroglobulin antibodies in most of the patients. Third, THRT was not offered to all subjects (SCH and overt hypothyroidism) so that we can evaluate the outcomes of treatment. Fourth, there is a paucity of collected data on the age of patients. More data are needed for better results of our analysis of median age. The study has not discussed the treatment of nonthyroid illness (NTI) like low T3 and low T4 with thyroid hormone, as the availability of guidelines is scarce.

## Conclusions

The CKD and thyroid disorders are independently highly prominent medical entities having high prevalence; hence it is essential to evaluate the physiological interactions of the thyroid in relation to renal disease. The present study shows a significant number of patients with CKD having a low level of T3 and T4. SCH was observed in an insignificant number of CKD patients of which females were more prevalent as compared to males. Biochemical overt hypothyroidism was also observed in some patients. So in this study, we conclude that chronic renal failure is a state of thyroid hypofunction as there was a higher prevalence of SCH and clinical hypothyroidism in cases of CKD. The severity of thyroid hypofunction increases with a progressive decrease in GFR.
